# Establishment of a gastric cancer cell line with high microsatellite instability, OCUM‐13, derived from Borrmann type‐2 primary tumor

**DOI:** 10.1002/cam4.5403

**Published:** 2022-11-02

**Authors:** Yurie Yamamoto, Go Masuda, Shuhei Kushiyama, Koji Maruo, Gen Tsujio, Tomohiro Sera, Atsushi Sugimoto, Sadaaki Nishimura, Kenji Kuroda, Shingo Togano, Tomohisa Okuno, Masaichi Ohira, Masakazu Yashiro

**Affiliations:** ^1^ Molecular Oncology and Therapeutics Osaka Metropolitan University Graduate School of Medicine Osaka Japan; ^2^ Cancer Center for Translational Research Osaka Metropolitan University Graduate School of Medicine Osaka Japan; ^3^ Department of Gastroenterological Surgery Osaka Metropolitan University Graduate School of Medicine Osaka Japan

**Keywords:** cell line, gastric cancer, microsatellite instability

## Abstract

Gastric cancer (GC) with microsatellite instability (MSI) has been reported to be sensitive to immunotherapy, however some of GC cases with MSI remain resistant to immunotherapy. Cancer cell lines showing MSI might be useful for the analysis of mechanisms of immunotherapy, while only a few GC cell lines with MSI are available so far. In this study, we established a unique GC cell line with MSI, OCUM‐13, from a primary GC with abundant tumor‐infiltrating lymphocytes. MSI assay indicated that OCUM‐13 cells as well as the primary tumor showed a band shift in more than 3 of 5 microsatellite loci, suggesting that OCUM‐13 did have high MSI. The subcutaneous inoculation of OCUM‐13 cells into mice performed tumor formation. Insulin‐like growth factor 1 receptor inhibitor decreased the growth of OCUM‐13 cells. The newly established cell line with MSI, OCUM‐13, might be useful for the analysis of cancer therapy for GC with MSI.

## 1 INTRODUCTION

Gastric cancer (GC) is one of the leading causes of cancer‐related deaths in the world.^1^ Because of the remaining poor prognosis of GC patients of advanced stage, the development of a new treatment strategy has been urgently desired.

Recently, GC has been classified on the basis of pathologic and macroscopic features.^2^ Currently, GC has been classified into 4 subtypes, including Epstein–Barr virus (EBV), microsatellite instability (MSI), genomic stability, and chromosomal instability, in accordance with a molecular biologic viewpoint.^3^

High MSI (MSI‐H) GC which observed in around 6%–8% of GC cases appears to show good sensitivity to immunotherapy^4^ because of high expression levels of tumor neoantigens.^5^ However, some cases of GC with MSI‐H remain resistant to immunotherapy.^6^ To improve the therapeutic outcome of MSI tumors, it is necessary to analyze the resistant mechanisms against immunotherapy and develop new effective therapies based on the biological characteristics using GC cells with MSI. MSI‐positive GC cell line might be useful to help clarify the mechanisms and develop effective therapies, there are still only a few reports of the establishment of GC cell lines with MSI has been available worldwide, so far.^7^, ^8^ In this study, we report on the establishment of a new high‐MSI GC cell line, OCUM‐13, which might contribute to the elucidation of the therapeutic mechanisms of GC with MSI‐H to immunotherapy.

## 2 MATERIALS AND METHODS

### 2.1 Patient and cell culture

A new GC cell line, OCUM‐13, was derived from a primary gastric tumor of a 62‐year‐old female in 2013. The primary gastric tumor was excised under aseptic conditions, and minced with forceps and scissors. Primary culture was initiated in May 2013. Pieces of the tumor were cultivated in 10 ml of Dulbecco's modified Eagle's medium (D‐MEM; Fujifilm Wako Pure Chemical Corporation, Osaka, Japan) with fetal bovine serum (FBS; Nichirei Bioscience, Inc.), 0.5‐mM sodium pyruvate (Sigma) and penicillin–streptomycin solution (Fujifilm Wako Pure Chemical Corporation). The adherent cell lines were found in the tumor tissue culture and were designated OCUM‐13. Adherent OCUM‐13 cells were detached and collected with trypsin (Fujifilm Wako Pure Chemical Corporation). OCUM‐13 cells were maintained for more than 24 months and passaged for more than 100 generations. We concluded that we had successfully established a new cell line based on the following references.^9^, ^10^ In this case the immortalization of isolated cells was not necessary for OCUM‐13 cells. All experiments were performed with mycoplasma‐free cells. This study was approved by the Osaka Metropolitan University Ethics Committee (reference number 912, 924, and 4391). Informed consent was obtained from the patient. This study has been conducted according to the principles of the declaration of Helsinki.

### 2.2 Tumorigenicity

Cell suspension containing 1 × 10^7^ OCUM‐13 cells were inoculated subcutaneously into BALB/c nude mice (Oriental Kobo, Osaka, Japan), and after 6 weeks, the tumor incidence was determined. The subcutaneous tumors were fixed in 10% formalin for paraffin sectioning. Animal experiments were performed in compliance with the guidelines of Ethics Committee.

### 2.3 Growth kinetics

The doubling time of OCUM‐13 cells was determined as follows. Briefly, suspensions of 1.0 × 10^4^ OCUM‐13 cells were incubated in 24‐well dishes with 1 ml D‐MEM containing 10% FBS. The number of cancer cells was counted every 24 h using Coulter Counter Z2 (BECKMAN COULTER). The doubling times were determined from the growth curve.

### 2.4 Chromosome analysis

Cells were karyotyped using a standard air‐dried method. They were analyzed using trypsin G banding.

### 2.5 Short Tandem Repeat analysis (STR)

In order to exclude cross‐contamination of cell lines, STR profiling was performed using the services provided by JCRB Cell Bank (Osaka, Japan).

### 2.6 RT‐PCR

Total cellular RNA was extracted from OCUM‐13 with RNeasy Plus Mini Kit, (QIAGEN). Next, cDNA was synthesized from 1 μg of RNA using ReverTra Ace qPCR RT Master Mix (TOYOBO). The cDNA was then subjected to 32 cycles of PCR with each primer on a thermal cycler using AmpliTaq Gold 360 cDNA polymerase (Thermo Fisher Scientific). The PCR conditions were as follows: annealing, 60°C × 30 sec.; extension, 72°C × 60 sec.; and final incubation, 72°C × 7 min.

### 2.7 MSI assay

Examination for MSI was performed using the service provided by BML using an ABI 3130xl Genetic Analyzer and The PowerPlex 4C Matric Standard (Promega). OCUM‐13 cells, primary tumor, and normal tissue in paraffin sections of the patient were analyzed. Six primer marker sets, NR‐21, BAT‐26, BAT‐25, NR‐24, MONO‐27, and Penta C, were used for MSI assay.

### 2.8 Growth inhibition assay

Five molecular target drugs, picropodophyllotoxin (Sigma Aldrich), sorafenib (Santa Cruz), bevacizumab (Amgen Inc.), ramucirumab (Cyramza), and cetuximab (Merck Serono) were used. Growth‐effect of the molecular target drugs on OCUM‐13 cell were examined using MTT assay (Dojindo, Kumamoto, Japan).

### 2.9 Immunohistochemistry

Immunohistochemical determination of IGF1R expression in the primary tumor tissue of the patient from which OCUM‐13 was derived was performed according to our previous study (Yamamoto Y et al. Anticancer Res. 2019 Dec;39[12]:6645–6652. doi: 10.21873/anticanres.13879. PMID: 31810929). Primary anti‐ IGF1R antibody (1:200, Bioss Antibodies, USA) was used in this study.

## 3 RESULTS

### 3.1 The primary gastric tumor and the establishment of a new gastric cancer cell line, OCUM‐13

**FIGURE 1:**
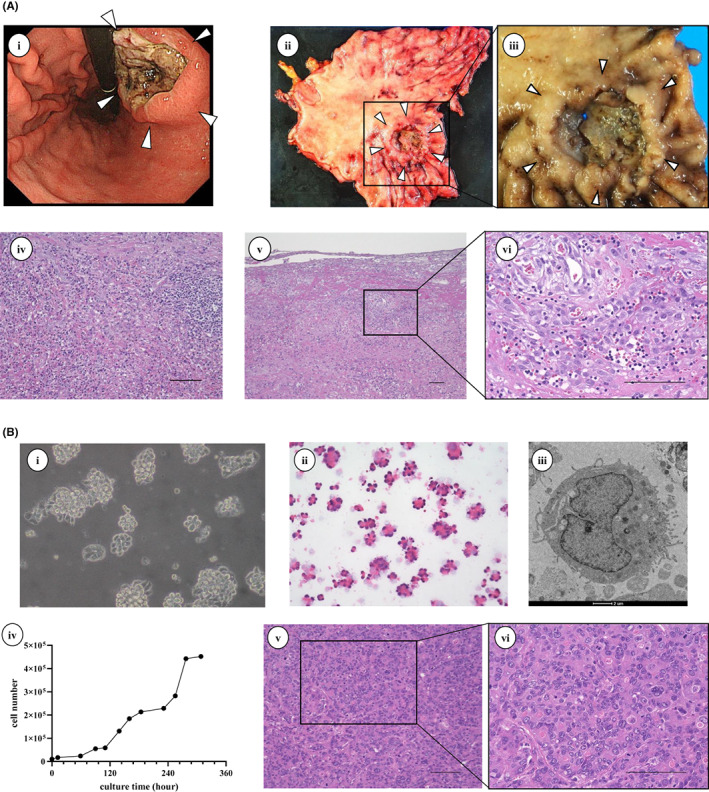
(A) Morphologic findings of primary gastric tumor and OCUM‐13 cells. A, Primary gastric tumor. i, Gastric endoscopy indicated that gastric tumor showed Bormann type 2. ii and iii, Primary tumor taken by distal gastrectomy. iv, v, and vi, Histologic findings of gastric tumor. The primary tumor was poorly differentiated adenocarcinoma with tumor‐infiltrating lymphocytes. Bar, 100 μm. B, OCUM‐13 cells. i, Phase‐contrast photomicrography of living OCUM‐13 cells. Most cells were adhered in paving stones shapes. ii, H&E staining of OCUM‐13 cells. iii, Electron micrograph of OCUM‐13 cells. iv, Growth curve of OCUM‐13 cells. v and vi, Histological images of subcutaneous tumors in mice by H&E staining. Moderate induction of stromal cells was observed, as was the tumor in the primary site. Bar, 100 μm.

Upper gastrointestinal endoscopy revealed that the primary gastric tumor showed a macroscopic Bormann‐type 2 tumor (Figure 1‐i). The resected stomach specimen showed an advanced 5‐cm‐diameter GC tumor localized at the posterior wall of the gastric body (Figure 1‐ii and ‐iii). The histopathologic findings of the primary tumor showed poorly differentiated adenocarcinoma (Type 2, por1, pT4s, med, infb, ly1, v1, pPM0, pDM0, pN0) with abundant tumor‐infiltrating lymphocytes (TILs) (Figure 1‐iv, ‐v, ‐vi). A new GC cell line, OCUM‐13, was successfully established from the primary tumor of the patient with GC. The immortalization of isolated cells was not necessary for OCUM‐13 cells. STR analysis indicated that the percentage of matched loci of OCUM‐13 cells in the STR profile databases was less than 70% (Figure S1). OCUM‐13 cells grew mainly in clusters and adhered to form paving stones (‐i). OCUM‐13 cells showed various‐sized nuclei which eccentrically‐located polarity outside of cells by hematoxylin and eosin (H&E) staining (Figure 1B‐ii). Figure 1B‐iii showed the shape of OCUM‐13 cells by electron microscopy. The doubling time of OCUM‐13 cells was 34 hours (Figure 1B‐iv). The subcutaneous inoculation of 1.0 × 10^7^ OCUM‐13 cells into mice resulted in 100% tumor formation (8/8). Histological examination of the subcutaneous tumor by HE staining showed features similar to those of the primary tumor, with moderate stromal cell induction. (Figure 1B‐v and ‐vi).

### 3.2 Chromosome analysis

**FIGURE 2:**
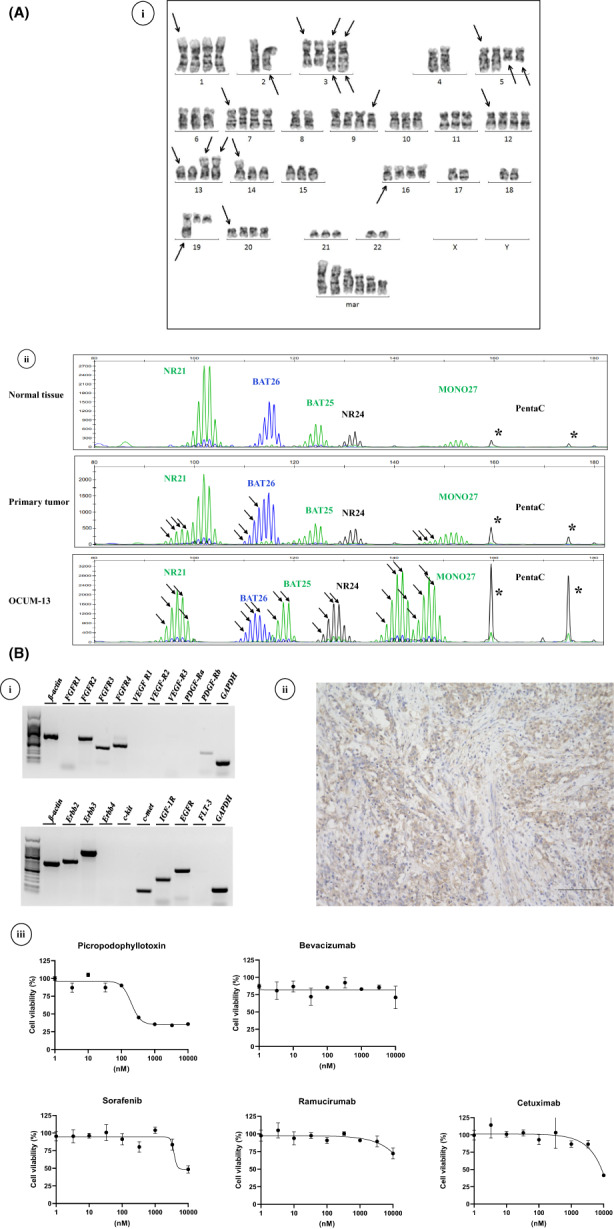
(A) Genetic analysis and expression of growth factor receptors. A, Chromosome analysis and microsatellite instability. i: G‐banding karyotype. The representative karyotype of OCUM‐13 was −X, ‐X, +1, −2, add(2)(q21), +3, der(3)del(3)(p13p21)add(3)(q27)×2, −4, +5, add(5)(q11.2)×2, +7, −8, +del(9)(p21), +12, Supplement Material 2. Yamamoto Y. *et al*. ‐2/2‐+13, add(13)(p11.2)×2, add(14)(p11.2), +add(16)(q22), ‐17, ‐18, add(19)(q13. 1‐13. 3), +20, ‐22, +6mar.^15^ The arrows indicate rearranged chromosomes. ii, MSI status. OCUM‐13 cells showed band shifts in all 5 MSI markers, NR‐21, BAT‐26, BAT‐25, NR‐24, and MONO‐27. The primary gastric tumor showed a band shift in 3 of 5 MSI markers. Arrows show band‐shifts. Penta C, a human identification marker, showed same fragment sizes among normal tissue, primary tumor tissue, and OCUM‐13 (asterisks). (B) Growth factor receptors and growth kinetics. i, mRNA expression of *FGFR2, FGFR3, FGFR4, PDGF‐Rb, Erb2, Erb3, c‐met, IGF‐1R,* and *EGFR*. ii, IGF1R expression in the primary tumor. Bar; 100 μm. iii, Effect of PPP, sorafenib, bevacizumab, ramucirumab, and cetuximab on the proliferation of OCUM‐13 cells. Growth of OCUM‐13 cells was significantly decreased by PPP. The IC50 of PPP was 196 nM. Sorafenib, bevacizumab, ramucirumab and cetuximab did not decrease proliferation of OCUM‐13 cells.

Chromosomal abnormalities were identified in 20 of 20 metaphase spreads examined. The number of chromosomes of OCUM‐13 cells ranged from 69 ~ 75, with a modal number of 75 (Figure 2Ai).

### 3.3 MSI

OCUM‐13 cells showed a novel band shift in all 5 microsatellite loci, NR‐21, BAT‐26, BAT‐25, NR‐24, and MONO‐27 (Figure 2A‐ii). OCUM‐13 cells were thus defined as having MSI‐H. The primary gastric tumor also showed a novel band shift in 3 of 5 MSI markers, and defined as having MSI‐H. Penta C, a human identification marker, indicated that normal tissue, primary tumor tissue, and OCUM‐13 have same fragment sizes, suggesting that normal tissue, primary tumor tissue, and OCUM‐13 might be the same individual.

### 3.4 Expression of Growth Factor Receptors in OCUM‐13 cells

mRNA expressions of FGFR2, FGFR3, FGFR4, PDGF‐Rb, Erb2, Erb3, c‐met, IGF1R, and EGFR were observed in OCUM‐13 cells, but those of FGFR1, VEGF‐R1, VEGF‐R2, VEGF‐R3, PDGF‐Ra, Erb4, c‐kit and FLT‐3 were not (Figure 2B‐i). IGF1R was expressed on cancer cells in the primary original tumor from which OCUM13 was derived. (Figure 2B‐ii).

### 3.5 Effects of molecular target drugs on the proliferation of OCUM‐13 cells

The IGF‐1R inhibitor, picropodophyllotoxin (PPP), significantly decreased the growth of OCUM‐13 cells. The half‐maximal inhibitory concentration (IC_50_) value of OCUM‐13 by PPP was 196 nM. However, sorafenib, bevacizumab, ramucirumab, and cetuximab did not inhibit the proliferative potential of OCUM‐13 (Figure 2B‐iii).

## 4 DISCUSSION

In this study, we newly established a GC cell line, OCUM‐13, from the primary tumor of a GC patient. OCUM‐13 cells showed adhesive growth and had a paving‐stone‐like shape, similar to many previously reported GC cell lines. Interestingly, the nuclei of cultured OCUM‐13 cells eccentrically‐located polarity outside of cells by H&E staining. OCUM‐13 cells injected subcutaneously into mice as xenografts achieved 100% tumor formation. The xenograft tumors histologically showed poorly differentiated adenocarcinomas, which was similar to the original human primary tumor from which OCUM‐13 cells were derived. These findings suggested that OCUM‐13 cells might reflect the histologic characteristics of primary tumors. OCUM‐13 cells might be useful for characterization of GC.

Interestingly, both OCUM‐13 cells and primary gastric tumor showed a band shift in more than 3 of 5 MSI markers, which suggested that OCUM‐13 cells, as well as original tumor, represented MSI‐H status. OCUM‐13 cells might possess similar MSI characteristics to primary tumor which was biologically classified into MSI subtype. In contrast, no familial history of cancer, such as Lynch syndrome, was found in the patient. These findings indicate that OCUM‐13 cells were established from a sporadic GC with MSI.

The histological findings of the gastric tumor demonstrated adenocarcinoma with tumor‐infiltrating lymphocytes (TILs) which is one of characteristic histologic features of MSI‐H tumors. Previous reports indicated that TILs might be associated with an adaptive immune response to tumor growth and with improved prognosis for GC, and that GC patients with MSI‐H might have better prognosis and higher levels of TIL in compared with GS patients with MSI‐low or stable.^11^ Kwon M. et al. reported that scanty stroma and high TIL were preferentially identified in responding patients.^12^ Since the primary tumor of this patient showed medullary growth with few stroma and high TILs, immunotherapy using PD‐1 blockade might be effective for this patient. It has been reported that patients with high tumor mutation burden (TMB) (TMB ≥10 mut/MB) have a higher probability of MSI‐H and a better response to immunotherapy.^13^ Sporadic MSI‐positive GCs, which account for 6–8% of GC,^14^ have shown to respond well to immunotherapy.^15^ MSI status has been highlighted as one of the key markers in determining the treatment strategy for cancer immunotherapy in various types of cancer^16^, ^17^; however, the response rate in patients with MSI‐positive GC remains less than 60%.^16^ Because MSI‐positive GC cell line might be necessary to clarify the mechanisms of immunotherapy, OCUM‐13 showing MSI‐H might be a useful cell line for the elucidation of the mechanism of immunotherapy resistance in MSI‐H GC.

OCUM‐13 cells expressed *IGF1R* mRNA by RT‐PCR analysis. The IGF1R inhibitor, PPP, significantly inhibited the proliferation of OCUM‐13 cells. These findings suggest that *IGF1R* is one of the driver genes of OCUM‐13 cells. In addition, IGF1R was expressed in the primary tissue of the patient from which OCUM13 was derived, suggesting that OCUM‐13 inherited the mimics of the original tumor and that IGF1R inhibitor might be effective for the original patient.

Since cross‐contamination is a common occurrence when establishing cell lines,^18^ we performed STR profiling of OCUM‐13 cells to investigate the possibility of cross‐contamination. The STR profile of OCUM‐13 cells did not correspond to any STR profiles of cells from the JCRB Cell Bank, indicating that OCUM‐13 is a unique GC cell line. Currently, there are 33 GC cell lines registered in the cell banks of ATCC, DSMZ, JCRB, and RIKEN. Among the 33 GC cell lines, 11 cell lines were established from a GC primary tumor, and the other 22 cell lines were established from a metastatic lesion of GC patient.

In conclusion, we have established a new MSI‐H GC cell line, OCUM‐13, from the primary tumor of a patients with GC. OCUM‐13 will play a useful role in future studies on immunotherapy, especially for GCs with MSI.

## AUTHOR CONTRIBUTIONS

**Yurie Yamamoto:** Methodology (equal); writing – original draft (equal). **Go Masuda:** Methodology (equal); resources (equal). **Shuhei Kushiyama:** Methodology (equal); resources (equal). **Koji Maruo:** Formal analysis (equal); investigation (equal); resources (equal). **Gen Tsujio:** Investigation (equal); methodology (equal); resources (equal). **Tomohiro Sera:** Investigation (equal); methodology (equal). **Atsushi Sugimoto:** Investigation (equal); methodology (equal). **Sadaaki Nishimura:** Investigation (equal); methodology (equal). **Kenji Kuroda:** Investigation (equal); methodology (equal). **Shingo Togano:** Investigation (equal); methodology (equal). **Tomohisa Okuno:** Investigation (equal); methodology (equal). **Masaichi Ohira:** Writing – review and editing (equal).

## CONFLICT OF INTEREST

The authors declare that they have no known competing financial interests in this paper.

## Supporting information


Figure S1

Appendix S1

Appendix S2

## Data Availability

All relevant data are within the manuscript and its Supporting Information file.

## References

[1] Smyth EC , Nilsson M , Grabsch HI , van Grieken NC , Lordick F . Gastric cancer. Lancet. 2020;396(10251):635‐648. 32861308 10.1016/S0140-6736(20)31288-5

[2] Japanese classification of gastric carcinoma: 3rd English edition. Gastric Cancer. 2011;14(2):101‐112. 21573743 10.1007/s10120-011-0041-5

[3] Comprehensive molecular characterization of gastric adenocarcinoma. Nature. 2014;513(7517):202‐209. 25079317 10.1038/nature13480 PMC4170219

[4] Chao J , Fuchs CS , Shitara K , et al. Assessment of Pembrolizumab therapy for the treatment of microsatellite instability‐high gastric or gastroesophageal junction cancer among patients in the KEYNOTE‐059, KEYNOTE‐061, and KEYNOTE‐062 clinical trials. JAMA Oncol. 2021;7(6):895‐902. 33792646 10.1001/jamaoncol.2021.0275 PMC8017478

[5] Agarwal P , Le DT , Boland PM . Immunotherapy in colorectal cancer. Adv Cancer Res. 2021;151:137‐196. 34148613 10.1016/bs.acr.2021.03.002

[6] Puliga E , Corso S , Pietrantonio F , Giordano S . Microsatellite instability in Gastric Cancer: Between lights and shadows. Cancer Treat Rev. 2021;95:102175. 33721595 10.1016/j.ctrv.2021.102175

[7] Yao Y , Tao H , Kim JJ , et al. Alterations of DNA mismatch repair proteins and microsatellite instability levels in gastric cancer cell lines. Lab Invest. 2004;84(7):915‐922. 15133479 10.1038/labinvest.3700117

[8] Yoon K , Lee S , Han TS , et al. Comprehensive genome‐ and transcriptome‐wide analyses of mutations associated with microsatellite instability in Korean gastric cancers. Genome Res. 2013;23(7):1109‐1117. 23737375 10.1101/gr.145706.112 PMC3698504

[9] Meditz K , Rinner B . Establishment of tumor cell lines: from primary tumor cells to a tumor cell line. In: Kasper C , Charwat V , Lavrentieva A , eds. Cell Culture Technology. Springer International Publishing; 2018:61‐73.

[10] Fredebohm J , Boettcher M , Eisen C , et al. Establishment and characterization of a highly tumourigenic and cancer stem cell enriched pancreatic cancer cell line as a well defined model system. PLoS One. 2012;7(11):e48503. 23152778 10.1371/journal.pone.0048503 PMC3495919

[11] Kim KJ , Lee KS , Cho HJ , et al. Prognostic implications of tumor‐infiltrating FoxP3+ regulatory T cells and CD8+ cytotoxic T cells in microsatellite‐unstable gastric cancers. Hum Pathol. 2014;45(2):285‐293. 24331841 10.1016/j.humpath.2013.09.004

[12] Kwon M , An M , Klempner SJ , et al. Determinants of response and intrinsic resistance to PD‐1 blockade in microsatellite instability‐high gastric cancer. Cancer Discov. 2021;11(9):2168‐2185. 33846173 10.1158/2159-8290.CD-21-0219

[13] Lee KW , Van Cutsem E , Bang YJ , et al. Association of tumor mutational burden with efficacy of Pembrolizumab±chemotherapy as first‐line therapy for gastric cancer in the phase III KEYNOTE‐062 Study. Clin Cancer Res. 2022;28(16):3489‐3498. 35657979 10.1158/1078-0432.CCR-22-0121

[14] Le DT , Durham JN , Smith KN , et al. Mismatch repair deficiency predicts response of solid tumors to PD‐1 blockade. Science. 2017;357(6349):409‐413. 28596308 10.1126/science.aan6733 PMC5576142

[15] Rodriquenz MG , Roviello G , D'Angelo A , Lavacchi D , Roviello F , Polom K . MSI and EBV positive gastric cancer's subgroups and their link with novel immunotherapy. J Clin Med. 2020;9(5):1427. 32403403 10.3390/jcm9051427 PMC7291039

[16] Ratti M , Lampis A , Hahne JC , Passalacqua R , Valeri N . Microsatellite instability in gastric cancer: molecular bases, clinical perspectives, and new treatment approaches. Cell Mol Life Sci. 2018;75(22):4151‐4162. 30173350 10.1007/s00018-018-2906-9 PMC6182336

[17] Polom K , Marano L , Marrelli D , et al. Meta‐analysis of microsatellite instability in relation to clinicopathological characteristics and overall survival in gastric cancer. Br J Surg. 2018;105(3):159‐167. 29091259 10.1002/bjs.10663

[18] MacLeod RA , Dirks WG , Matsuo Y , Kaufmann M , Milch H , Drexler HG . Widespread intraspecies cross‐contamination of human tumor cell lines arising at source. Int J Cancer. 1999;83(4):555‐563. 10508494 10.1002/(sici)1097-0215(19991112)83:4<555::aid-ijc19>3.0.co;2-2

