# Supplementary material for: Establishment of a gastric cancer cell line with high microsatellite instability, OCUM‐13, derived from Borrmann type‐2 primary tumor
Source: Cancer Med. 2022 Nov 2;12(5):6016–22. doi: 10.1002/cam4.5403 (PMC10028156; doi:10.1002/cam4.5403)
Supplement: Supplementary file 1 — Figure S1 [file CAM4-12-6016-s001.pdf]

13/Aug./2021

JCRB Cell Bank

National Institute of Biomedical Innovation
7-6-8 Saito-Asagi, Ibaraki, Osaka, Japan
TEL : +81-72-641-9851
FAX : +81-72-641-9859

Cell Authentication Report (KBN0794)

Client:

Yamamoto Yurie
Department of Molecular Oncology and Therapeutics,
Osaka City University Graduate School of Medicine
TEL : 06-6645-3936
E-mail : m9563702@med.osaka-cu.ac.jp

Sample Information

Sample Number : 1

1) Cell No. : KBN0794-01 (Cell Name : OCUM13)

Peak report (Cell No. : KBN0794-01)

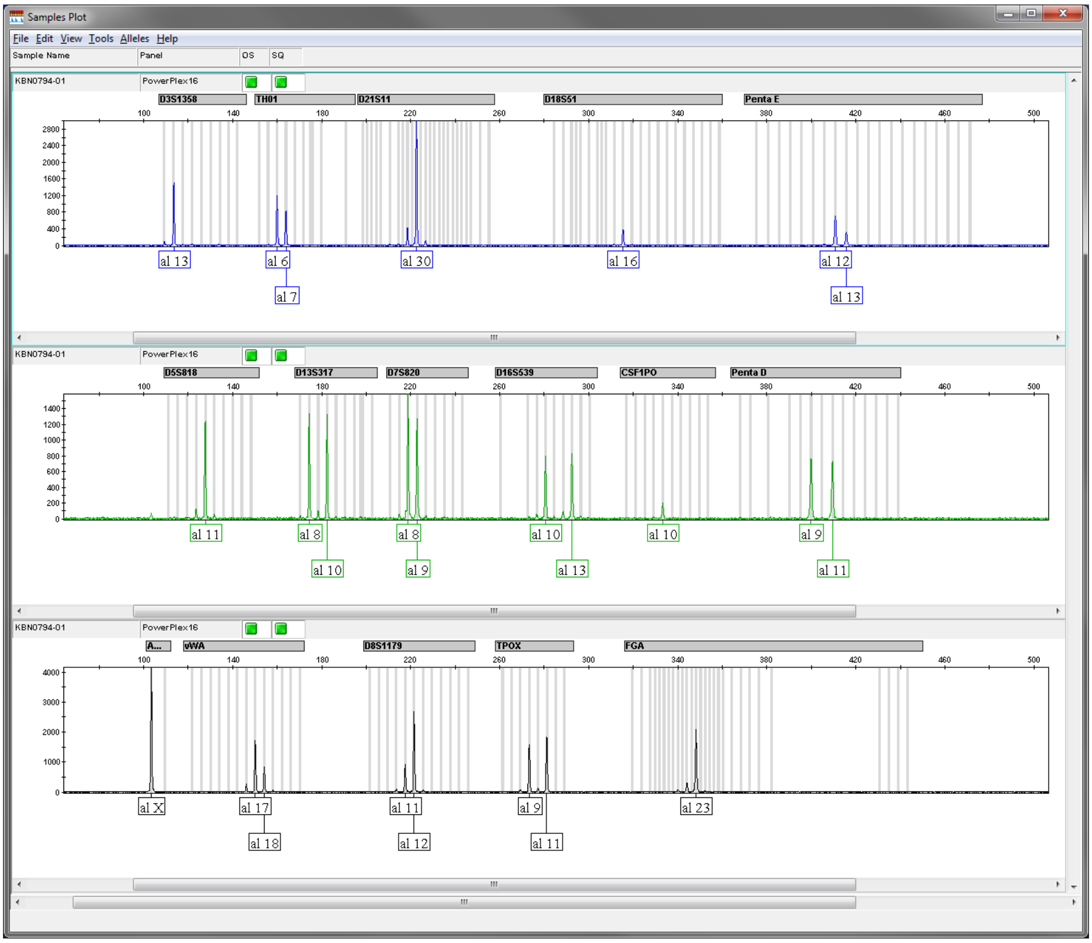

STR Profile (Cell No. : KBN0794-01)

D3S1358	TH01	D21S11	D18S51	Penta E
13	6,7	30	16	12,13

D5S818	D13S317	D7S820	D16S539	CSF1PO	Penta D
11	8,10	8,9	10,13	10	9,11

AM	vWA	D8S1179	TPOX	FGA
X	17,18	11,12	9,11	23

Comparison with database (Cell No. : KBN0794-01)

Cell No.	Cell Name	EV	D5S818	D13S317	D7S820	D16S539	vWA	TH01	AM	TPOX	CSF1PO
**KBN0794-01**	**OCUM13**	**----**	**11**	**8,10**	**8,9**	**10,13**	**17,18**	**6,7**	**X**	**9,11**	**10**
RCB0784	FCP-S2H	0.667	11	8,10	11	9,10	17,18	7,9	X	11	9,10
CRL-7846	Hs 571.T	0.667	11	10,11	8,9	11,12	18	6,9.3	X	8,11	10
IFO50315	RMG-I	0.611	12	8,12	11	9,10	17,18	6,7	X	11	10
IFO50316	RMG-II	0.611	12	8,12	11	9,10	17,18	6,7	X	11	10
JCRB0822	NUGC-3	0.611	11	10,11	8,9	10,11	18	6,8	X	8,9	13
JCRB1042	BSL2KA	0.611	11	8,9	8,12	10,11	14,17	6,9	X	9,11	11,12
JCRB1062	JHH-1	0.611	11	8,10	9	9,13	17	7	X	8,11	12,13
JCRB1476	HCC-1954-Luc	0.611	11	8,9	10,11	9,11	18,19	6,7	X	8,9	10
JCRB3016	XP2KA	0.611	11	8,13	8	10,11	17,18	9	X	8,11	10,12
RCB0444	RERF-LC-AI	0.611	11	12	8,12	10	17	7	X	11	10

Summary (Cell No. : KBN0794-01)

It was confirmed that the cell (Cell No.: KBN0794-01, Cell Name: OCUM13) was not corresponding to the cell of the database of JCRB Cell Bank. STR patterns other than the examined cell could not be detected.
