# Supplementary material for: Establishment of a gastric cancer cell line with high microsatellite instability, OCUM‐13, derived from Borrmann type‐2 primary tumor
Source: Cancer Med. 2022 Nov 2;12(5):6016–22. doi: 10.1002/cam4.5403 (PMC10028156; doi:10.1002/cam4.5403)
Supplement: Supplementary file 2 — Appendix S1 [file CAM4-12-6016-s002.docx]

**Figure Legends**

**Figure 1**. **Morphologic findings of primary gastric tumor and OCUM-13 cells.**

**A, Primary gastric tumor**. **ⅰ,** Gastric endoscopy indicated that gastric tumor showed Bormann type 2. **ⅱ and ⅲ**, Primary tumor taken by distal gastrectomy. **ⅳ**, **ⅴ**, and **ⅵ**, Histologic findings of gastric tumor. The primary tumor was poorly differentiated adenocarcinoma with tumor-infiltrating lymphocytes. Bar, 100 µm. **B, OCUM-13 cells.** **ⅰ**, Phase-contrast photomicrography of living OCUM-13 cells. Most cells were adhered in paving stones shapes. **ⅱ**, H&E staining of OCUM-13 cells. **ⅲ**, Electron micrograph of OCUM-13 cells. **ⅳ**, Growth curve of OCUM-13 cells. **ⅴ and ⅵ**, Histological images of subcutaneous tumors in mice by H&E staining. Moderate induction of stromal cells was observed, as was the tumor in the primary site. Bar, 100 µm.

**Figure 2. Genetic analysis and expression of growth factor receptors.**

**A, Chromosome analysis and microsatellite instability. ⅰ:** G-banding karyotype. The representative karyotype of OCUM-13 was -X, -X, +1, -2, add(2)(q21), +3, der(3)del(3)(p13p21)add(3)(q27)×2, -4, +5, add(5)(q11.2)×2, +7, -8, +del(9)(p21), +12, +13, add(13)(p11.2)×2, add(14)(p11.2), +add(16)(q22), -17, -18, add(19)(q13. 1-13. 3), +20, -22, +6mar [15] The arrows indicate rearranged chromosomes. **ⅱ,** MSI status. OCUM-13 cells showed band shifts in all 5 MSI markers, NR-21, BAT-26, BAT-25, NR-24, and MONO-27. The primary gastric tumor showed a band shift in 3 of 5 MSI markers. Arrows show band-shifts. Penta C, a human identification marker, showed same fragment sizes among normal tissue, primary tumor tissue, and OCUM-13 (asterisks).

**B, Growth factor receptors and growth kinetics. ⅰ,** mRNA expression of *FGFR2, FGFR3, FGFR4, PDGF-Rb, Erb2, Erb3, c-met, IGF-1R,* and *EGFR*. **ⅱ,** IGF1R expression in the primary tumor. Bar; 100μm. **ⅲ,** Effect of PPP, sorafenib, bevacizumab, ramucirumab, and cetuximab on the proliferation of OCUM-13 cells. Growth of OCUM-13 cells was significantly decreased by PPP. The IC50 of PPP was 196 nM. Sorafenib, bevacizumab, ramucirumab and cetuximab did not decrease proliferation of OCUM-13 cells.
